# Integrated microbiomic and metabolomic dynamics of Yi traditional fermented liquor

**DOI:** 10.1016/j.fochx.2024.102016

**Published:** 2024-11-16

**Authors:** Hanqiao Liang, Zidong Zhu, Yong Fan, Jinghong Hu, Jiaqi Wu, Ziying Mu, Yang Li, Qin Wei, Chunmei Yang, Jing Tian, Shouqian Li

**Affiliations:** a Department of Biomedicine, Beijing city university, Beijing 100083, china; b The Eighth Medical Center of Chinese PLA General Hospital, Beijing 100700, China; c College of life sciences & food engineering, Key lab of aromatic Plant resources exploitation and utilization in sichuan higher education, Yibin university, Yibin 644000, china; d Guizhou Jinqianguo Biotechnology Co., Ltd., Next to Gongjia Bridge, Zhuchang Town, Guizhou Province Building B, Returning Migrant Workers Entrepreneurship Park, 551700, China

**Keywords:** Yi traditional fermented food, Fermentation, Metagenomics, Volatile flavor compounds, Metabolomics

## Abstract

This study examines the microbial community composition, metabolite characteristics, and the relationship between the two during the fermentation process of Yi traditional fermented liquor. Yi traditional fermented foods have a profound historical and cultural background, with significant ethnic characteristics. As a case in point, Yi traditional fermented liquor is typically prepared using local plants or traditional Chinese herbs as fermentation substrates and undergoes a lengthy fermentation process, resulting in a fermented beverage that is reputed to have beneficial effects on human health. These foods are not only characterised by a distinctive flavor profile, but are also perceived to possess certain health benefits in the context of traditional ethnic medicine and wellness practices. The community composition of bacteria and fungi was analyzed using 16S rRNA and ITS1 sequencing technologies, which revealed that microbial diversity was higher in the early stages of fermentation but gradually decreased as fermentation progressed. A total of 130 major volatile flavor compounds and 26 key metabolites were identified at different stages of fermentation. These included acids, sugars, amino acids and flavonoids, which significantly influence the flavor and nutritional value of the fermented products. The study indicates a significant correlation between specific microbial populations (such as yeasts) and key metabolites (such as flavonoids and amino acids). These findings emphasise the significance of the interplay between microbial communities and metabolites in shaping the quality and efficacy of fermented products. They offer a scientific foundation for optimizing traditional fermented food production processes.

## Highlights

• Microbial community structure are closely related at different fermentation stages.
• 130 major volatile flavor compounds were detected in the fermentation process.
• 26 key metabolites were identified affecting the sensory properties.
• The changes of microbial diversity and metabolites were closely related to fermentation stage.

## 1 Introduction

For thousands of years, the industrious and brave Chinese nation has developed a rich traditional food culture through continuous practice and refinement, forming the foundation of national reproduction and development. Yi is one of the numerous ethnic minorities in China, possess a rich and diverse traditional culture and dietary customs, with traditional fermented foods being an important component of their food culture. Such as sour soup, Mouding fermented tofu, Lunan pickled tofu, and black bean sauce. The Yi people, residing in the Yunnan-Guizhou Plateau, benefit from the area's unique climate and geographical conditions, which provide a wealth of medicinal resources. Jieben-fermented liquor, a Yi traditional fermented food with a long history and strong national characteristics, originated from the secret recipe of “Jieben immortal liquor” of the Suozhuo family in Bijie. The secret recipe, formulated in 1882 by the Yi people in Bijie, Guizhou province, has been passed down through generations. Utilizing a variety of rare natural plants, the recipe undergoes microbial fermentation in an ancient altar for over 300 days in a low-temperature, metal-free environment. This process allows the healthy ingredients in the plants to fully participate in microbial growth and metabolism, creating a fermentation liquid rich in specific small-molecule biologically active ingredients. The unique raw materials and processing methods endow it with various physiological functions, such as effectively inhibiting SARS-CoV-2 replication in vitro and reconstructing dysregulated intestinal flora (Fang et al., 2020; Wang et al., 2022).

Bijie, Guizhou province, located in the Wumeng Plateau, boasts a rich natural ecology and abundant biological resources. The fermentation process not only enhances efficacy but also improves drug acceptance and flavor. The fermented medicinal liquor primarily consists of honey, *Rosa roxburghii*, coconut, lemon, and leftover sweet seeds. *Rosa roxburghii,* belonging to the Rosaceae family and widely distributed in Guizhou, has roots, leaves, and fruits that stop diarrhea, relieve heat, and promote digestion. It is often used in traditional Chinese homology of medicine and food to treat various diseases, enhancing immunity and anticancer properties, reducing blood sugar, and providing antioxidant benefits (Chen et al., 2014; Westhuizen et al., 2008; Xu et al., 2014; Zhang et al., 2001), earning it the title “king of vitamin C". Due to the fruit's sour taste, fresh thorn pear fruit has low public acceptance, making deep processing essential. Biological fermentation reduces bitterness and introduces new active ingredients (Liu et al., 2023). Coconut, rich in coconut water, contains various nutrients such as sugar, amino acids, vitamins, and minerals, making it a natural medium for microbial fermentation (Fei et al., 2024). Rich in various carbohydrates (e.g., glucose, fructose, sucrose, and sorbitol), these carbohydrates provide carbon sources and amino acids for the growth of Acetobacter. Additionally, the vitamins, minerals, organic acids, and other ingredients in coconut water may aid in bacterial cellulose synthesis (Zhang et al., 2017). Coconut also enhances its nutritional properties due to its high concentrations of fat, antioxidants, and minerals such as iron, magnesium, and zinc (Mesquita et al., 2020). Lemon fruit is abundant in active compounds like organic acids, vitamin C, flavonoids, phenolic acids, and minerals. These compounds in lemons exhibit multiple activities, including antioxidant and anticancer effects, and can improve neurodegeneration and cognitive impairment (Zhu et al., 2023). Yuganzi is known for its rich polyphenols, which have a strong astringent taste initially. The seeds contain high concentrations of polyphenols (33 % of dry weight), flavonoids, and amino acids, providing excellent antioxidant properties and potential antifatigue, antihyperlipidemia, and antitumor benefits (Huang et al., 2023).

The metabolic activities of microbiota during food fermentation include proteolysis and glycolysis, which are crucial for producing volatile compounds and forming specific flavors (Zhao et al., 2022). Metabolomics strategies can identify and quantify numerous compounds in biological samples without bias (Want et al., 2007), helping us understand biochemical changes over time and processing (Capanoglu et al., 2008).

In 2019, traditional fermented Yi traditional fermented medicinal liquor was listed as a provincial intangible cultural heritage by the Guizhou Provincial Department of Culture. However, few studies have explored the relationship between metabolites and microflora in the main varieties of fermentation liquid. This study's novelty lies in analyzing changes in the substrate's chemical composition during fermentation and using nontargeted metabolomic analysis with GC-TOF-MS to understand metabolite level changes and evaluate the potential for producing valuable compounds. By employing redundancy analysis (RDA) and co-heatmap analysis at different fermentation stages, we were able to discern significant correlations between pivotal microorganisms and essential metabolites. The purpose of this study is to deepen the understanding of the important functional microbiome involved in the fermentation process of **Jieben fermented liquor**, clarify the role of the microbiome in flavor substances, and provide a theoretical basis for the development of traditonal fermented foods. The study fills gaps in existing research on fermented foods, especially traditional medicines.

## 2 Materials and methods

### 2.1 Preparation and sample collection

Jieben fermented liquor (JBFML) samples adopted low-temperature solid-state fermentation were provided by Guizhou Jinqianguo Biotechnology Co., Ltd., Bijie City, Guizhou Province, China, which holds the largest production scale in Guizhou Province. The JBFML samples were randomly selected at the fermentation stages corresponding to months 1 (A), 2 (B), 3 (C), 4 (D), 6 (E), 8 (F) and 10 (G). To ensure the accuracy and reliability of the results, five samples were randomly selected from various locations within the fermentation bottol, thereby providing a representative overview and reducing the potential for errors. All samples were transported to the laboratory on ice and stored at − 80 °C until further analysis.

### 2.2 Analysis of physicochemical

1. Determination of effective acidity: pH was measured using a calibrated pH meter (pH-3C, Rex Electric Chemical, China).
2. Determination of total solids: A handheld refractometer according to NY/T 2637–2014 was used to test the samples.
3. Determination of titratable acid content: according to method II of GB 12456–2021 for sample testing.
4. Determination of DPPH radical scavenging activity: Take 100 μL of each sample solution and add 100 μL of DPPH ethanol solution (0.04 mg/100 mL). Mix thoroughly and incubate for 30 min in the dark at room temperature. Measure the absorbance at 517 nm. Use an equal volume of ethanol to replace the DPPH solution as the blank group and water to replace the sample solution as the control group. Calculate the scavenging rate from the formula.

$$\text{Clearance rate}=1-\frac{A_{S}-A_{C}}{A_{0}}$$

A_0_ — Absorbance of 100 μL anhydrous ethanol + 100 μL DPPH solution;As — Absorbance of 100 μL sample solution + 100 μL DPPH solution;Ac — Absorbance of 100 μL sample solution + 100 μL anhydrous ethanol.

### 2.3 Microbial communities analysis

#### 2.3.1 DNA extraction

DNA from the fermented liquor samples was extracted using the E.Z.N.A. Soil DNA Kit (Omega Bio-tek, Inc., USA) and the extraction method was described in the kit instructions. DNA samples were stored at −20 °C for subsequent experiments.

#### 2.3.2 DNA amplification and sequencing

Universal primers 338F (5’-ACTCCTACGGGAGGCAGCAG-3′) and 806R (5’-GGACTACHVGGTWTCTAAT-3′) were used to amplify the V3-V4 region of the bacterial 16S rRNA gene. For the ITS1 region of the fungal rRNA gene, ITS1 (5’-CTTGGTCATTTAGAGGAAGTAA-3′) and ITS2 (5’-TGCGTTCTTCATCGATGC-3′) primers were used for amplification. An 8 bp barcode sequence was appended to the 5′ end of all upstream and downstream primers to differentiate between samples.

The PCR was carried out on a Mastercycler Gradient (Eppendorf, Germany) using 25 μL reaction volumes, containing 12.5 μL 2× Taq PCR MasterMix (Vazyme Biotech Co.,Ltd., China), 3 μL BSA(2 ng/μl), 1 μL Forward Primer (5 μM), 1 μL Reverse Primer (5 μM), 2 μL template DNA, and 5.5 μL ddH_2_O. Cycling parameters were 95 °C for 5 min, followed by 28 cycles of 95 °C for 45 s, 55 °C for 50 s and 72 °C for 45 s with a final extension at 72 °C for 10 min. The PCR products were purified using a Agencourt AMPure XP Kit (Beckman Coulter, Inc., USA).

The purified PCR amplifiers were sent to Illumina Miseq/Nextseq 2000/Novaseq 6000 for deep sequencing analysis. The raw data was divided into different samples according to the barcode sequence. Use Pear (Zhang et al., 2014) (v0.9.6) software to filter and splice raw data. The sequences were removed from consideration if they contained ambiguous bases N, and cut out the parts with low quality score (≤ 20) in the sequences. During splicing, the minimum overlap setting was 10 bp, and the *p*-value setting was 0.0001. After splicing, Vsearch (Rognes et al., 2016) (v2.7.1) software was used to remove sequences with length less than 230 bp and removed the chimeric sequence by uchime (Edgar et al., 2011) method according to the Gold (Bacteria 16S) or Unite (Fungus ITS) Database. Qualified sequences were clustered into operational taxonomic units (OTUs) at a similarity threshold of 97 % use Uparse (Edgar, 2013) algorithm of Vsearch (v2.7.1) sofware. The BLAST (Ye et al., 2006) tool was used to classify all OTU representative sequences into different taxonomic groups against corresponding Database and e-value threshold was set to1e-5.

### 2.4 GC–MS non-target metabolome analysis

Approximately 25 ± 1 mL of the JBFML was mixed with a steel ball. Then, 1000 μL of pre-cooled extraction solvent (methanol = 3:1, containing internal ribonucleol) was added. The mixture was processed using a grinding machine at 35 Hz for 4 min, followed by ultrasonic treatment in an ice water bath for 5 min, repeated three times. The samples were placed in a − 40 °C refrigerator to stand for one hour. Afterward, the samples were centrifuged at 4 °C for 15 min at 12,000 rpm, and 100 μL of supernatant was transferred to a fresh 1.5 mL tube. For the preparation of a QC (Quality Control) sample, 20 μL from each sample was combined. After evaporation in a vacuum concentrator, 30 μL of methoxyamine hydrochloride (20 mg/mL in pyridine) was added, and the mixture was incubated at 80 °C for 30 min. Then, 40 μL of BSTFA reagent (1 % TMCS, *v*/v) was added for derivatization at 70 °C for 1.5 h. The samples were gradually cooled to room temperature, and 5 μL of FAMEs (in chloroform) was added to the QC sample. Finally, all samples were analyzed using gas chromatography coupled with a time-of-flight mass spectrometer.

GC-TOF-MS (Kind et al., 2009) analysis was performed using an Agilent 7890 gas chromatograph coupled with a time-of-flight mass spectrometer. And detailed information is provided in the supplementary materials S1 (Table S1). Raw data analysis, including peak extraction, baseline adjustment, deconvolution, alignment and integration, was finished with Chroma TOF (V 4.3×，LECO) software and LECO-Fiehn Rtx5 database was used for metabolite identification by matching the mass spectrum and retention index. Finally, the peaks detected in less than half of QC samples or RSD>30 % in QC samples was removed.

### 2.5 LC-MS non-target metabolome analysis

The samples were thawed on ice. After 30 s vortex, 250 μL aliquot of individual samples were transferred to an Eppendorf tube and nitrogen dried. 500 μL of extract solution (methanol/water = 3:1, precooled at −40 °C, containing internal standard) were added to the samples. After 30 s vortex, the samples were sonicated for 10 min in icewater bath. Then the samples were centrifuged at 12000 rpm for 15 min at 4 °C. The supernatant was carefully filtered through a 0.22 μm microporous membrane, and each sample was taken and pooled as QC samples, which stored at −80 °C until the UHPLC- MS analysis (Wang et al., 2011).

The UHPLC separation was carried out using an EXIONLC System (Sciex). The mobile phase A was 0.1 % formic acid in water, and the mobile phase B was acetonitrile. The column temperature was set at 40 °C. The auto-sampler temperature was set at 4 °C and the injection volume was 2 μL. A Sciex QTrap 6500+ (Sciex Technologies), was applied for assay development. Typicalion source parameters were: IonSpray Voltage: +5500/−4500 V, Curtain Gas: 35 psi,Temperature: 400 °C, Ion Source Gas 1:60 psi, Ion Source Gas 2: 60 psi, DP: ± 100 V. And detailed information is provided in the supplementary materials S1 (Table S2). Sciex Analyst Work Station Software (Version 1.6.3) was employed for MS data acquisition and processing. MS raw data (.wiff) files were converted to the TXT format using MSconventer. In-house R program and database were applied to peak detection and annotation.

### 2.6 Data analysis

First of all, metabolite feature is detected in <20 % of experimental samples or detected in <50 % of QC samples, it is removed from data analysis (Dunn et al., 2011). Then the missing values of raw data were filled up by half of the minimum value. In addition, internal standard normalization method was employed in this data analysis. Finally, features with RSD >30 % should be removed from the subsequent analysis. The resulted three-dimensional data involving the peak number, sample name, and normalized peak area were fed to R package metaX (The R package metaX is a software package used for PCA and OPLS-DA analysis) (Wen et al., 2017) for principal component analysis (PCA) and orthogonal projections to latent structures-discriminate analysis (OPLS-DA) (Andersen et al., 2012). Principal component analysis (PCA) showed the distribution of origin data. In order to obtain a higher level of group separation and get a better understanding of variables responsible for classification, supervised projections to latent structures-discriminate analysis (OPLS-DA) were applied (Triba et al., 2015). Based on the projections to latent structures-discriminate analysis (OPLS-DA), a loading plot was constructed, which showed the contribution of variables to difference between two groups. To refine this analysis, the first principal component of variable importance in the projection (VIP) was obtained. The VIP values exceeding 1.0 were first selected as changed metabolites. In step 2, the remaining variables were then assessed by Student's *t*-test (Q-value <0.05), variables were discarded between two comparison groups. In addition, commercial databases including KEGG (http://www.kegg.jp) and MetaboAnalyst (http://www.metaboanalyst.ca/) was utilized to search for the pathways of metabolites.

## 3 Results and discussion

### 3.1 Physicochemical properties

**Fig. 1:**
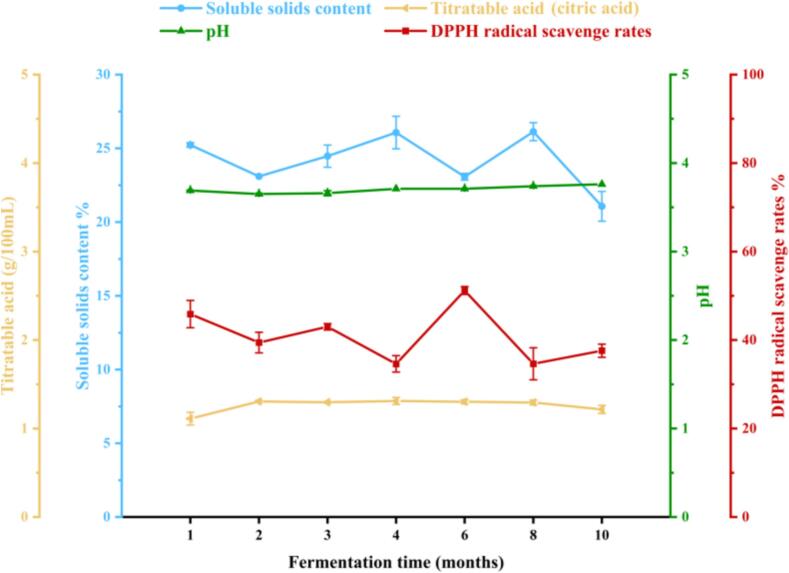
Changes in physicochemical properties during Jieben fermentation

Based on the data from Table S3 and trends in Fig. 1, the division of fermentation time into phases is based on the observed trends in key fermentation parameters: pH, titratable acidity, soluble solid content, and DPPH radical scavenging ability. Each phase represents a distinct period of change or stability in these parameters, which reflects underlying biochemical and microbial processes occurring during fermentation. In the initial decline phase (1–2 months), the pH decreased significantly from 3.69 to 3.65, titratable acidity increased rapidly from 1.1122 g/100 mL to 1.3062 g/100 mL, and soluble solid content decreased from 25.23 % to 23.10 %. In the stable fluctuation phase (2–6 months), the pH fluctuated slightly between 3.65 and 3.71 but remained stable overall. During this period, titratable acidity (2–8 months) ranged from 1.2950 to 1.3115 g/100 mL with minor fluctuations, and soluble solid content (2–8 months) remained relatively stable, ranging from 23.07 % to 26.13 %. In the later rising phase (6–10 months), the pH increased significantly to 3.76. During the slight decline phase for titratable acidity (8–10 months), the acidity decreased slightly to 1.2163 g/100 mL, though the change was not significant. In the significant decline phase for soluble solid content (8–10 months), the content dropped significantly to 21.07 %. Regarding DPPH radical scavenging ability, the scavenging rate dropped sharply from 45.86 % in the first month to 34.62 % by the 4th month. It then rose to 51.20 % in the 6th month, which was the highest point in the cycle. Subsequently, the rate fell sharply to 34.63 % by the eighth month. Finally, during the steady recovery phase (8–10 months), the scavenging rate gradually rose to 37.60 % by the 10th month.

### 3.2 Microbial diversity during the fermentation

#### 3.2.1 Microbiome analysis of bacterium

**Fig. 2:**
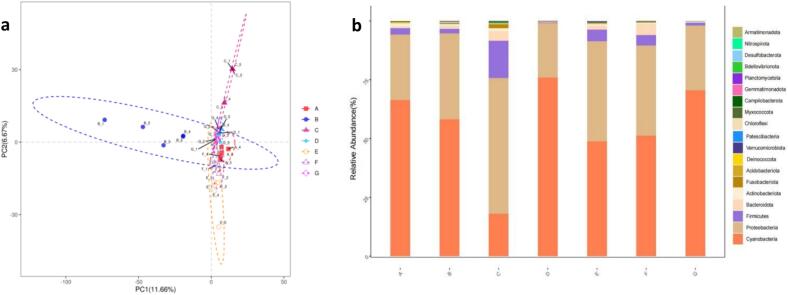
PLS-DA analysis (a) and distribution of bacterial community structure succession at the phylum level (*n* = 5) (b).

The analysis of alpha diversity showed that the bacterial diversity was considerably higher than that of fungi. The bacteria in the samples were sequenced and 46,177,727 raw data were obtained. A total of 4,488,113 valid sequences were obtained with the V3-V4 region of the 16S rRNA gene. The sequences were subjected to OTU (Operational Taxonomic Unit) clustering at 97 % similarity. Following screening, a total of 3373 OTUs were generated, and the number was optimised to 3044 bacterial OTUs after the draw-leveling process. Partial Least Squares Discrimination Analysis (PLS-DA) revealed that bacterial compositions at different fermentation stages varied at the OTU level (Fig. 2a). Significant differences in bacterial structure were observed between the 2nd, 3rd, and 6th months of fermentation compared to other times. The three main dominant phyla were *Cyanobacteria* (average abundance 55.6 %, abundance range 18.08 %–76.04 %), *Proteobacteria* (average abundance 36.2 %, abundance range 2.3 %–57.74 %), and *Firmicutes* (average abundance 4.5 %, abundance range 1.15 %–15.08 %). The combined relative abundance of these phyla exceeded 96 % in all fermentation samples, indicating that the bacterial community structure of fermented liquor exhibited a high degree of stability at different fermentation stages. PLS-DA analysis indicated a close relationship between bacterial composition in the fermented drug and fermentation time. The histogram of bacterial community composition at the phylum level (Fig. 2b) showed that *Cyanobacteria* and *Proteobacteria* were the most dominant among the 20 bacterial groups. The relative abundance of *Proteobacteria* in the 3rd month of fermentation was significantly higher than in the other six groups (*p* < 0.05), while Cyanobacteria's relative abundance was significantly lower (*p* < 0.05), with the highest abundance in the 4th month. These results suggest that the bacterial structures of Yi traditional fermented medicinal liquor vary significantly with the fermentation stage.

**Fig. 3:**
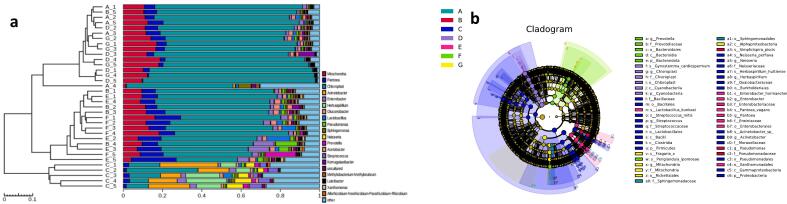
Bacterial species cluster analysis (a) and LEfSe analysis (b) at the family level (Kruskal-Wallis and rank test α < 0.05; LDA score > 4.00).

To investigate differences in bacterial composition and identify species with significant abundance differences between groups (i.e., biomarkers of the fermentation process), cluster analysis (CA) (Fig. 3a) and Linear discriminant analysis Effect Size (LEfSe) (Fig. 3b) were conducted on seven groups of fermentation drug samples at the family level. The CA results showed that the bacterial composition of the samples in the 3rd month (C) of fermentation differed significantly from the other samples. The bacterial compositions of the samples from the 2nd(B), 6th(E), and 8th(F) months of fermentation clustered together, while the bacterial compositions of the samples from the 1st(A), 4th(D), and 10th(F) months of fermentation also clustered together. LEfSe analysis was performed to compare the differences in fungal communities various stages of fermentation (Fig. 3b). Sample 3rd month (C) of fermentation, which differed significantly from the other samples, was mainly enriched by Clostridia, Bacillaceae, Acinetobacter (containing *Acinetobacter* sp), Neisseria (e.g., *Neisseria perflava*), Herbaspirillum (including *Herbaspirillum huttiense*), Streptococcus (specially *Streptococcus mitis*), which accounting for 2.07 % of the total bacterial group is the highest one.

**Fig. 4:**
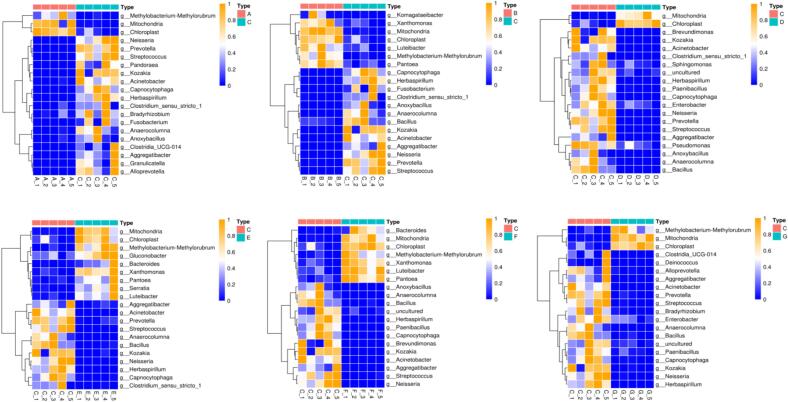
Heatmap based on the bacterial composition at the genus level of 20 top significantly changed between C group and other groups in total

Using group C as a benchmark, the other sample groups were compared with group C, and a Wilcoxon test was performed (Fig. 4). The relative abundance of *Mitochondria* and *Chloroplast* was higher in the other sample groups compared to group C, while group C had higher levels of *Acinetobacter*.

#### 3.2.2 Microbiome analysis of fungi

**Fig. 5:**
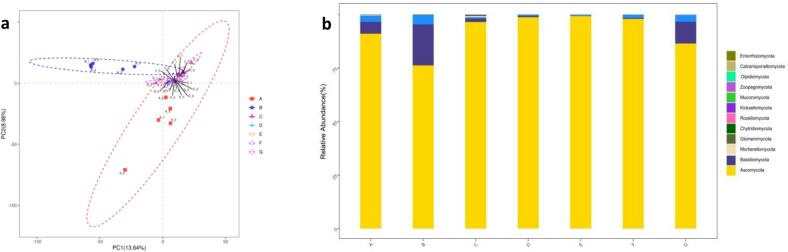
PLS-DA analysis (a) and distribution of fungal community structure succession at the phylum level (*n* = 5) (b).

The fungal samples were subjected to sequencing, resulting in the acquisition of 4,617,727 raw data points. A total of 2,179,590 high-quality sequences, with a length of 200–260 bp, were obtained for the ITS1 region of the fungal ITS gene. The sequences were then subjected to OTU (Operational Taxonomic Unit) clustering at 97 % similarity. Following screening, a total of 4800 OTUs were generated, with the number subsequently optimised to 3538 bacterial OTUs following draw leveling. Partial Least Squares Discrimination Analysis (PCA) revealed differences in fungal composition at the OTU level across fermentation stages (Fig. 5a). Significant differences in fungal structure were observed between the 1st and 2nd months and other fermentation times. The dominant fungal phyla in the fermentation process of fermented liquor were *Ascomycota* and *Basidiomycota*. The mean abundance of *Ascomycota* was 92.3 %, with a range of 76.3 % to 99.3 %. In contrast, the mean abundance of *Basidiomycota* was 5.4 %, with a range of 0.05 % to 5.5 %. The *Ascomycetes* phylum continued to exert a dominant influence throughout the fermentation process. After a period of two months, the relative abundance of *Ascomycetes* exhibited a decline, from 91 % to 76.3 %. Concurrently, the relative abundance of *Stachybacteria* demonstrated an increase, from 5.5 % to 19.2 %. It is notable that the relative abundance of *Ascomycetes* remained above 86.5 % at all other fermentation stages. PLS-DA indicated that fungal composition in fermented liquor was closely related to fermentation time. Fungal community composition at the phylum level (Fig. 5b) showed *Ascomycota* as the dominant phylum among the 13 fungal taxa, with little variability across samples (*p* > 0.05). *Ascomycota* was also the dominant fungal group in all types of white liquor (Wang, 2018). *Basidiomycota* followed, with significantly higher abundance (*p* < 0.05) in the first two months and the last month of fermentation. The fungal structure of Yi traditional fermented medicinal liquor varied significantly with the fermentation stage.

**Fig. 6:**
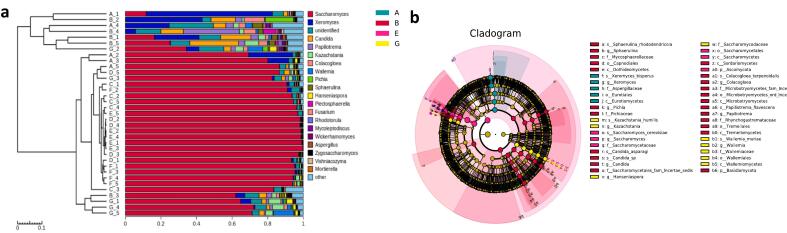
Fungal species cluster analysis (a) and LEfSe analysis (b) at the family level (Kruskal-Wallis and rank test α < 0.05; LDA score > 4.00).

To explore differences in microbial composition and identify species with significant abundance differences between groups (i.e., biomarkers of the fermentation process), CA (Fig. 6a) and LEfSe analysis (Fig. 6b) were performed at the genus level for four groups of fermented samples. The CA results showed that the fungal composition of samples in the 1st and 2nd months of fermentation differed significantly from the other samples. The fungal compositions of samples from the 3rd, 4th, 6th, and 8th months of fermentation clustered together, while the fungal compositions of samples from the 10th month clustered individually. LEfSe analysis was performed to compare the differences in fungal communities various stages of fermentation (Fig. 6b). Sample 2nd month (B) of fermentation, which differed significantly from the other samples, was mainly enriched by Sphaerulina (containing *Sphaerulina rhododendricola*), Saccharomycetales (involving *Pichia* and *Candida asparagi*), Colacogloea (including *Colacogloea terpenoidalis*), Papiliotrema (specially *Papiliotrema flavescens*), which accounting for 2.77 % of the total bacterial group is the highest one.

#### 3.2.3 Comparison of bacterial and fungal changes during fermentation

**Fig. 7:**
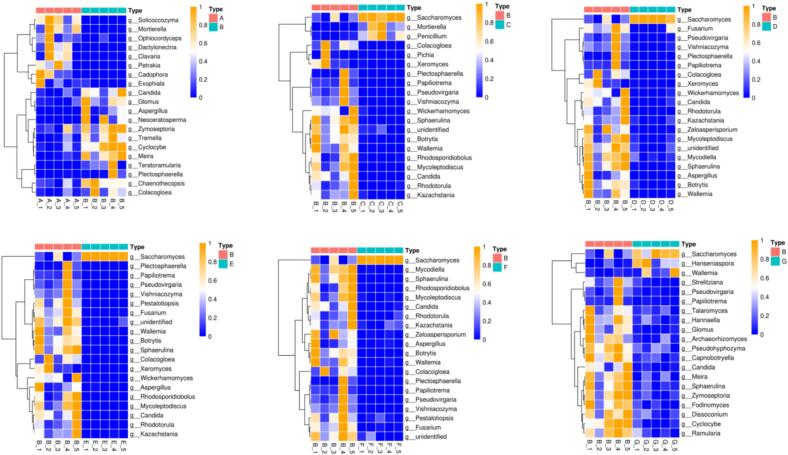
Heatmap based on the fungal composition at the genus level of 20 top significantly changed between B group and other groups in total

The analysis of alpha diversity showed that the bacterial diversity was considerably higher than that of fungi (Table S4 ∼ S5). Principal component analysis and CA indicated that the microbial composition of the first two months of fermentation differed significantly from the other samples. The samples in group B clustered better, and using group B as a benchmark, the other sample groups were compared with it, and a Wilcoxon test was performed (Fig. 7). The relative abundance of *Saccharomyces* was higher (*p* < 0.05) in the other sample groups compared to group B. Conversely, the abundance of *Xeromyces* was higher in sample B (*p* < 0.05). *S. cerevisiae* plays a key role in liquor fermentation due to its excellent alcohol tolerance (Djeni et al., 2020), resistance to high temperatures, and strong competitiveness (Lin, 2023). The dominance of *S. cerevisiae* in the fermentation process may lead to an increase in the alcohol concentration of medicinal liquors.

### 3.3 Volatile flavor compound analysis

**Fig. 8:**
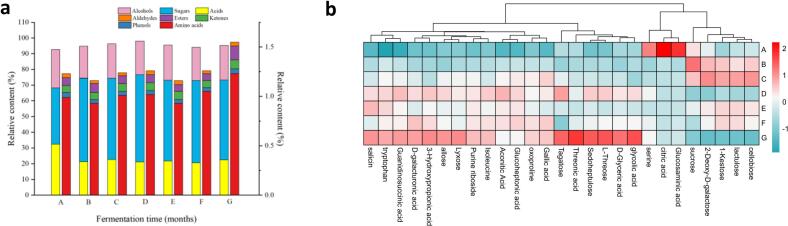
Relative content of volatile flavor compound types (a). Heatmap of changes in major volatile flavor compounds during fermentation of JBFML, red: high concentration, white: medium Heatmap of changes in major volatile flavor compounds during fermentation of JBFML, red: high concentration, white: medium concentration, and blue: low concentration (b). (For interpretation of the references to colour in this figure legend, the reader is referred to the web version of this article.)

During fermentation, 130 major volatile flavor compounds were detected, including 48 acids, 27 sugars, 19 amino acids, 18 alcohols, 3 ketones, 2 phenols, 2 aldehydes, 2 esters, and 9 other volatile compounds. Specifically, 113, 118, 117, 118, 117, 117 and 115 volatile compounds were identified in samples A, B, C, D, E, F, and G, respectively. The number of volatile compounds remained relatively stable during the fermentation. As shown in Fig. 8a, sugars were the major volatile compounds identified, accounting for 38.78 %, 53.74 %, 50.14 %, 47.54, 47.01, 46.73, and 46.57 %, respectively. Alcohols and acids decreased rapidly in the second month, while sugar content increased significantly. Specifically, D-allulose, gibberellose, D-grape heptose, and sucrose were the main sugar compounds in the fermented liquor. Two new sugar compounds, erythrulose and raffinose, were produced after fermentation, along with two new acid compounds, 3,4-dihydroxybenzoic acid and aminosuccinic acid, and two new amino acid compounds, L-homoserine and tryptophan. The original acids in the solution included citric acid, L-malic acid, and succinic acid, with citric acid being the most abundant at a maximum of 16.54 %. In the later stages of fermentation, citric acid and ethyl isovalerate content decreased slightly. Alcohols began to increase slowly in the third month, with sugar alcohols, particularly glycerol, having the highest content (Yang, 2017; Zhu et al., 2019). Glycerol can remove the strange odor of aldehydes in liquor during fermentation and enhance the product's sweetness. It plays a significant role in balancing taste and smoothness in medicinal liquors. Small amounts of amino acids, ketones, phenols, and aldehydes were also detected during fermentation.

The contents of acids and alcohols were higher in the first month of fermentation, while the total amino acids and esters increased in the tenth month. The total sugar content increased significantly from the second month onward, indicating the production of a large amount of amino acid metabolites, esters, and sugars during the fermentation of Yi traditional fermented liquor. Studies have shown that amino acids enhance the sensory characteristics of food products (Lei et al., 2019). Esters significantly affect the flavor, producing a unique floral and fruity aroma that greatly influences the flavor of Yi traditional fermented liquor (Dong et al., 2023; Zhu et al., 2020).

The volatile compound contents at different fermentation stages, categorized and displayed in Fig. 8b, showed that different categories of volatile compounds exhibited varying trends throughout the fermentation process. This suggests that fermentation time significantly affects the flavor of fermented liquor.

For an in-depth analysis, volatile compounds from different fermentation times were analyzed using partial least squares-discriminant analysis (OPLS-DA) to assess their differences (Consonni et al., 2011). The B,C,D,E,F samples were well differentiated by the identified volatile compounds, as shown in the OPLS-DA score plot (R^2^Y: 0.695, Q^2^: 0.601). Cross-validation analysis confirmed the model's reliability (R^2^ = 0.15, Q^2^ = −0.824). Based on the variable importance projections in the OPLS-DA model (VIP > 1.0, *p* < 0.05) (Pan et al., 2023), 43 volatile compounds were identified as key differential flavor compounds. These included 15 acids, 14 sugars, 6 amino acids, 2 nucleic acids, 1 alcohol, 1 ketone, and 4 other compounds.

### 3.4 Metabolite analysis

**Fig. 9:**
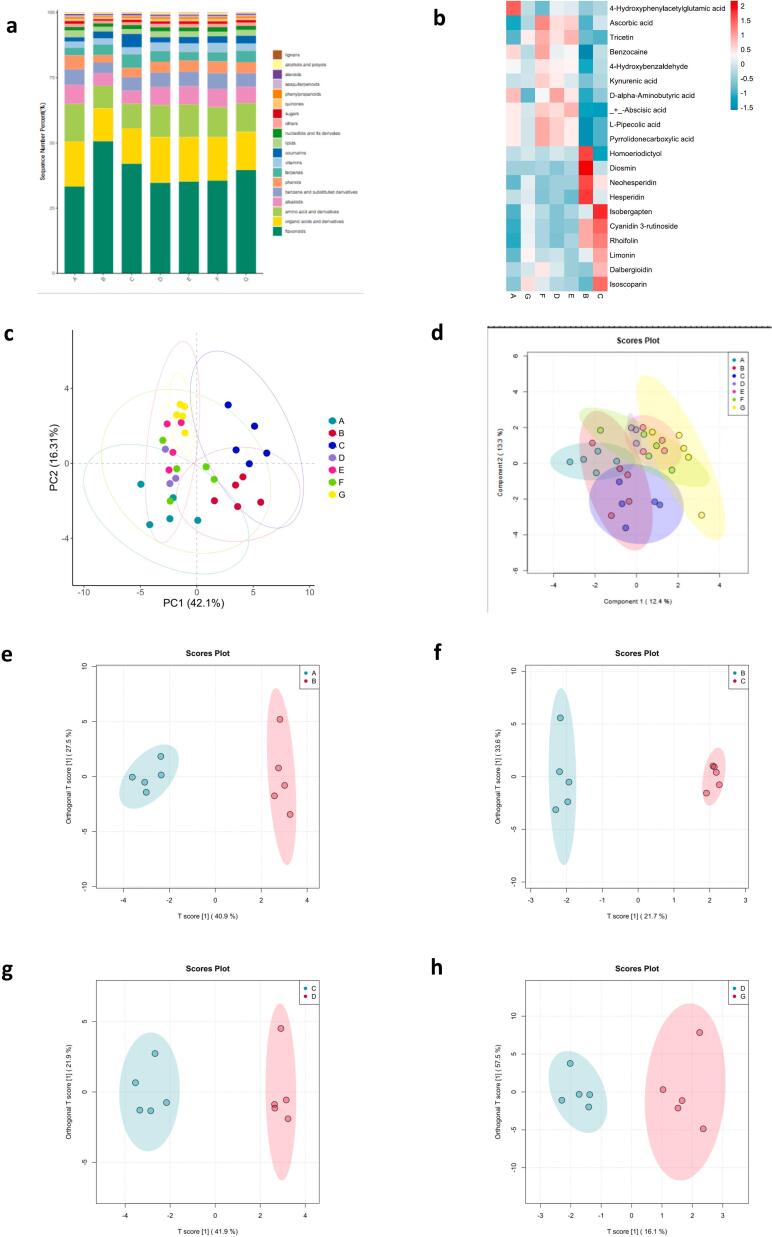
Comparison of the metabolites of fermention stages. (a) The content of metabolites. (b) key metabolite during fermention stages. Principal component analysis (PCA) and Orthogonal partial least squares-discriminant analysis (OPLS-DA) score plots of the metabolites (c: PCA, d: OPLS-DA). OPLS-DA score plots of metabolites in samples (e: 1 month vs. 2 month, f: 2 month vs. 3 month, g: 3 month vs. 4 month, h: 4 month vs. 10 month).

Metabolomics provides a comprehensive analysis of chemical constituents (Zhang et al., 2024), characterizing quality by qualitatively and quantitatively identifying thousands of metabolites, especially those undetectable by traditional methods (Wang et al., 2023). Flavonoids, organic acids, amino acids, and alkaloids were the main metabolite species, accounting for more than 70 % of the total (Fig. 9a).

During fermentation, 26 key metabolites with high or significant relative quantitative values were identified, including ten flavonoids, six organic acids, one lipids, phenols, coumarin and terpene (Fig. 9b). The results from previous microorganism studies showed significant changes during the first three months of fermentation, a critical period by the third month, with a slowing down of material changes from the fourth month onward. During the third month, the contents of N6-isopentenyladenosine and ribulose adenosine diphosphate increased several tens of times. Although the content gradually decreased in later stages, it remained several times higher than before fermentation at the end. The contents of indole ethanol aldehyde, N6-isopentenyladenosine, ribulose adenosine diphosphate, diethyl phosphorothioate, and ethyl 3,4,5-trimethoxyphenylacetic acid increased by a factor of 5–10 by the end of fermentation. Flavonoids accounted for the highest percentage of all samples at 35.17 %, with neohesperidin, mainly derived from lemon, increasing from 5.96 % to 8.86 %. Organic acids and amino acids accounted for the second and third highest percentages at 14.85 % and 12.35 %, respectively. However, their content decreased by 1.86 % and 2.58 %, respectively, as fermentation progressed. 4-Hydroxyphenylacetylglutamic acid had the highest content, decreasing from 5.38 % before fermentation to 3.56 %. Although terpenes and coumarins were initially low, their content doubled and changed significantly after fermentation. Alcohols and sugar alcohols decreased rapidly at the start of fermentation, then stabilized with minimal changes toward the end.

Metabolites at different fermentation times were analyzed using principal component analysis (PCA) and orthogonal partial least squares-discriminant analysis (OPLS-DA) score plots (Fig. 9c and d). The first two principal components accounted for 58.41 % of the total variance, with PC1 and PC2 contributing 42.10 % and 16.31 %, respectively. Based on the PCA score plots, four fermentation stages were identified: the first month as Stage a, the second month as Stage b, the third month as Stage c, and the period from the fourth to the tenth month as Stage d (Fig. 9 e–h).

Differential metabolites were identified with VIP > 1.00, *P* < 0.05, and FC > 2.00 or FC < 0.20 (Table S6). In total, 54 differential metabolites were found between A and B, with 53 up-regulated and 1 down-regulated. Notably, there was a significant increase in flavonoids, alkaloids, nucleotides, phenols, sugars, and terpenoids. Between B and C, 27 metabolites differed (24 upregulated and 3 downregulated), while 24 metabolites differed between C and D (14 up-regulated and 10 down-regulated). In contrast, only 10 differential metabolites (9 upregulated and 1 downregulated) were found between D and G.

Initial fermentation phase: The rapid increase in metabolites may be linked to heightened microbial growth and metabolic activity during fermentation. For instance, the rise in flavonoids and nucleotides could be associated with microbial metabolism and the breakdown of raw materials. Critical phase (third month): This phase marks a metabolic turning point, potentially due to changes in fermentation conditions or shifts in the microbial community. Significant increases in N6-isopentenyladenosine and ribulose adenosine diphosphate may be tied to energy metabolism during fermentation. Later fermentation stages: the slowing down of material changes might result from the gradual depletion of nutrients or the microbial community entering a steady state. The accumulation and reduction of metabolites could be related to the stabilization of fermentation products and the deceleration of microbial activities.

### 3.5 Relationships between major microorganisms and major volatile flavor compounds and nonvolatile flavor compounds

**Fig. 10:**
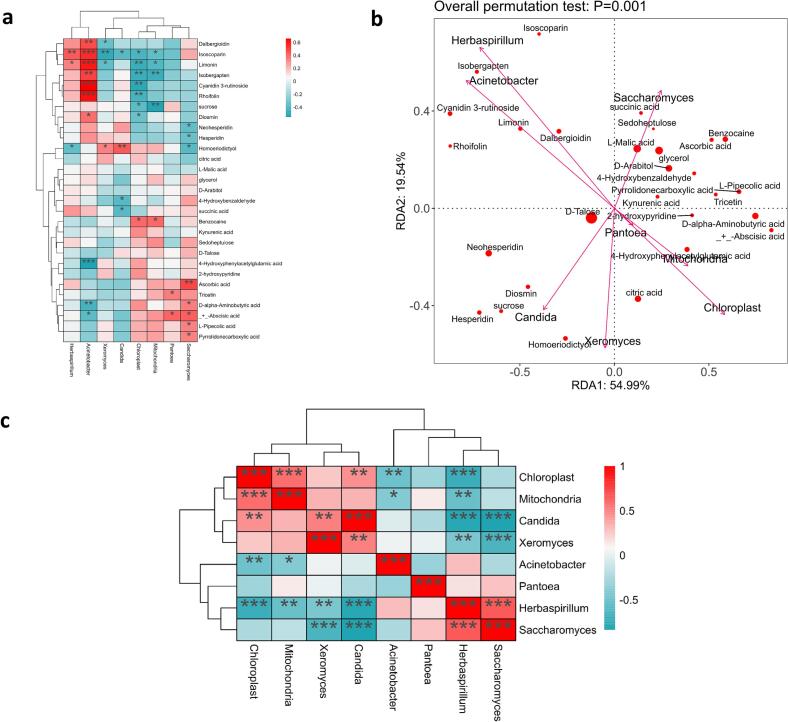
Co-heatmap of predominant microorganisms and major metabolites (A). Redundancy analysis (RDA) of predominant microorganisms and major metabolites (B).

Redundancy analysis (RDA) and co-heat maps revealed potential relationships between major microorganisms and metabolites (Figs. 10a and b). *Acinetobacter*, *Chloroplast*, *Mitochondria*, and *Saccharomyces* were linked to isoscoparin, limonin, isobenzophenone (Isobergapten), cyanidin 3-rutinoside, rhoifolin, and ascorbic acid. Isobenzylene, citrinin, cyanidin 3-rutinoside, and rhoifolin showed a strong positive correlation with *acidophilus* and a negative correlation with *chloroplasts*. *Mitochondria* were strongly correlated with isobenzophenone and sucrose. Additionally, *Saccharomyces cerevisiae* correlated with ascorbic acid, D-alpha-aminobutyric acid, abscisic acid, L-pipecolic acid, and pyrrolidonecarboxylic acid.

Correlation heatmaps (Fig. 10c) annotated the relationships between major microorganisms. *Herbaspirillum* showed strong negative correlations with *chloroplasts*, *mitochondria*, *Candida,* and *Xeromyces*. Conversely, *chloroplasts* and *mitochondria*, as well as *Herbaspirillum* and *S. cerevisiae*, exhibited strong positive correlations. *Candida* had a strong negative correlation with *C. herbicola* and *Saccharomyces cerevisiae*, but a positive correlation with *chloroplasts* and *Xeromyces cerevisiae*.

### 3.6 Metabolic pathways

**Fig. 11:**
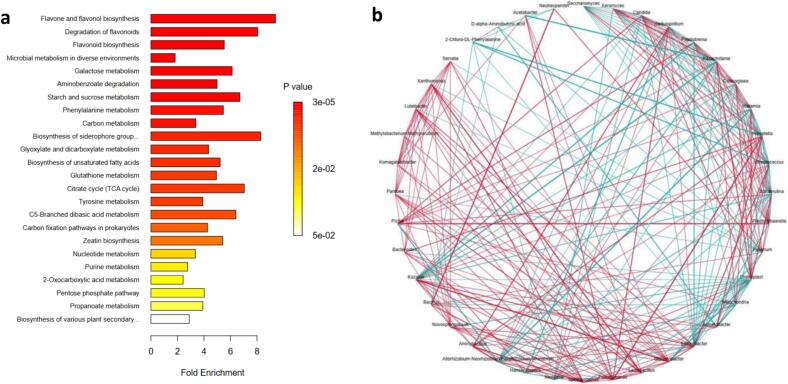
Differential metabolite pathway enrichment analysis (a) and network (b), showing the pathways that are related to the metabolites that change the most during fermentation.

Using the KEGG database (Kanehisa et al., 2023), an enrichment analysis of 24 key metabolic pathways was conducted (Fig. 11a), highlighting the flavonoid and flavonol biosynthesis pathways as the most distinct. In the fermentation process reveals the dynamic changes in key metabolic pathways and the interrelationships between metabolites, which significantly influence the quality and characteristics of the fermentation products. Notably, the biosynthetic pathway of flavonoids is significantly enriched, indicating an active synthesis of flavonoids such as neohesperidin, which confers important antioxidant properties to the fermented beverage. The activity of amino acid metabolism pathways and the decline in γ-aminobutyric acid may affect the flavor and mouthfeel of the wine. Within the metabolite network, the associations between neohesperidin and various organic acids and amino acids suggest that its synthesis or metabolism might be regulated by these components, thereby influencing the antioxidant characteristics and flavor of the wine. Furthermore, the positive correlation between *Saccharomyces cerevisiae* and the antioxidant ascorbic acid highlights its contribution to antioxidant activity. The association of Pichia manshurica with volatile compounds such as isoamyl alcohol and phenethyl alcohol indicates its role in enhancing fruit and floral aromas. The strong correlation of Lactobacillus with acetic acid and butyric acid underscores its significant impact on the acidity and flavor profile during fermentation. Overall, these metabolic pathways and microbial interactions not only enhance the flavor and sensory dimensions of the fermented beverage but also provide a scientific basis for optimizing fermentation processes and improving product quality. Key metabolites and major metabolic pathways linked to JIEBEN were identified (Fig. 11b). For volatile flavor compounds, pyruvate is converted to 2,3-butanediol. Phosphoenolpyruvate produces *L*-phenylalanine, which is then transformed into phenylethanol. The glycolytic pathway generates volatile flavor compounds like ethanol and acetic acid. The tricarboxylic acid cycle intermediate, 2-ketoglutarate, is metabolized by arginine and proline to form glyoxylatephosphoenolpyruvate (PEP), synthesizes *L*-phenylalanine and pyruvate, with *L*-phenylalanine subsequently producing L-tyrosine. Pyruvate and glycolytic pathways yield L-lactate. L-valine, L-isoleucine, and (*S*)-2-acetyl lactate are indirectly produced via pyruvate. The tricarboxylic acid cycle generates various organic acids, including malic acid, L-malic acid, and pyruvic acid. This cycle also produces amino acids such as L-glutamic acid, L-proline, and L-arginine. Vinic acid and citric acid are derived from pyruvate and tricarboxylic acid cycle intermediates. These pathway analyses align with observed trends in key metabolic levels.

## 4 Conclusion

Significant variations in bacterial and fungal community structures were noted at different fermentation stages, particularly in the early and third months. *S cerevisiae* was the dominant microorganism, impacting alcohol concentration due to its alcohol tolerance, high-temperature resistance, and strong competitiveness. In the fermentation process, the absence of ethanol and the high glycerol content in the final product can be attributed to several factors related to the metabolic adaptability of S *cerevisiae*. Under low oxygen conditions, yeast may shift from ethanol production to glycerol synthesis as a means to reoxidize NADH, thus maintaining redox balance without ethanol formation (Costenoble et al., 2000). Additionally, in response to high osmotic stress, where the fermentation medium exhibits elevated osmolarity, S *cerevisiae* produced glycerol for osmoregulation, leading to its accumulation in the fermentation products. Nutrient limitation, particularly nitrogen deficiency, can also induce a metabolic shift favoring glycerol synthesis over ethanol production. Moreover, metabolic regulatory factors, such as the upregulation of GPD1 and GPD2 genes encoding glycerol-3-phosphate dehydrogenase under high NADH reoxidation demands, contributed to increased glycerol yields. In summary, the phenomenon of high glycerol and no ethanol in fermentation products may be due to hypoxia, osmotic stress, nutrient limitation, and specific metabolic regulation, which collectively promoted glycerol synthesis at the expense of ethanol production (Jiang et al., 2018). Bacterial diversity surpassed fungal diversity, with multiple bacterial and fungal phyla identified. The bacterial community was primarily composed of the phyla Cyanobacteria, Ascomycetes, and Thick-walled bacteria (Fig. 2b), while the fungal community was predominantly represented by the phyla Ascomycetes and Stramenobacteria (Fig. 5b), suggesting that the bacterial community exhibited greater complexity. The PLSDA plots and bar charts (e.g., Figs. 2a, b, 5a, b) clearly demonstrated the differences in the diversity of the bacteria and the fungi. These results indicated that the diversity of the bacteria was high and that the phyla were more abundant, whereas the fungal community was relatively simple. This was further corroborated by the cluster analysis (CA) results, which demonstrated significant differences in the community composition of bacteria and fungi at different fermentation stages (Figs. 3a and 6a). The quantitative data on the number and relative abundance of OTUs supported this finding, indicating that bacterial diversity indices remained high across fermentation stages, whereas the diversity of fungi was lower and less variable. The aforementioned results were demonstrated through graphs and data to indicate that bacterial diversity was significantly higher than fungal diversity across different fermentation stages. This provides a scientific basis for understanding the microbial community dynamics during fermentation and optimizing the fermentation process. During fermentation, 130 volatile flavor compounds were detected. Sugars, the primary volatile compounds, increased significantly after the second month, while acids decreased in the second month and alcohols rose in the third month. Total amino acids and esters increased in the tenth month of fermentation, influencing sensory characteristics and flavor. During the fermentation process in the tenth month, the metabolism of amino acids not only provided essential precursors for energy metabolism but also directly promoted the formation of various esters, thereby enriching the aroma and flavor of the fermentation products (Kruis et al., 2017). Acetyl-CoA was produced from glutamate through the TCA cycle, which reacted with ethanol to form ethyl acetate with apple and pear aromas, enhancing the fruity characteristics (Verstrepen et al., 2003). Acetyl-CoA generated from the decomposition of alanine further produced ethyl acetate and isoamyl acetate with banana-like aroma, adding a sweet fruitiness. Phenylalanine metabolism resulted in the formation of phenylethanol, which combined with acetic acid to form phenylacetic acid ethyl ester with floral notes, enhancing the richness and complexity of the overall aroma. Valine and leucine, as branched-chain amino acids, generated ketone bodies and acetyl-CoA through the branched-chain amino acid metabolic pathway. Notably, acetyl-CoA produced from leucine reacted with alcohols to form hexyl acetate with apple and strawberry aromas, adding a refreshing layer to the product's aroma (Lee et al., 2018). Intermediate products from isoleucine reacted with short-chain alcohols to generate ethyl acetate and butyl acetate with pear, apple, and pineapple aromas, further enhancing the layering of tropical fruit scents (Ma et al., 2020). The coupling of amino acid and ester metabolism brought about a rich array of aroma layers and complex fruity flavors, improving the sensory quality of the fermented products.Volatile compounds varied throughout fermentation, with fermentation time significantly impacting the flavor of medicinal liquors. Metabolite changes occurred in four stages, with the most significant changes in the first three months, making the third month a critical period. Flavonoids, neohesperidin, organic acids, and amino acids varied significantly, affecting the characteristics of the fermented product. Specific microorganisms showed significant correlations with key metabolites: *acidophilus* positively correlated with various metabolites, chloroplasts negatively correlated with some metabolites, and mitochondria closely correlated with others. *S. cerevisiae* was associated with metabolites like ascorbic acid. The interactions between microorganisms are complex, and their dynamic equilibrium greatly influences the fermentation process and metabolite accumulation.

The present study revealed significant associations between key microorganisms and metabolites during the fermentation of traditional Yi fermented liquor. This was achieved through the use of redundancy analysis (RDA) and correlation heat maps, which provided a deeper understanding of the relationship between microorganisms and flavor and functional components. For example, *Saccharomyces cerevisiae* was the dominant yeast species involved in ethanol and glycerol production during fermentation, contributing to the wine's flavor profile. *Acetobacter* species oxidised ethanol to produce acetic acid, giving the fermented wine a sour taste. Meanwhile, *Lactobacillus* species produced lactic acid and, in combination with other bacteria, aromatic esters, adding complexity to the wine's aroma. *Xeromyces* species promoted the production of volatile esters in the later stages of fermentation, contributing to the development of fruity and floral aromas. Furthermore, the active metabolism of mitochondria in the late fermentation stage facilitated the accumulation of antioxidants, including ascorbic acid and flavonoids, which provided a crucial safeguard for the functionality of traditional Yi fermented liquor. These findings not only enhance our understanding of the complex interactions between microorganisms and metabolites, but also provide a scientific basis for optimizing the fermentation process and improving product quality.

## CRediT authorship contribution statement

**Hanqiao Liang:** Writing – original draft, Methodology, Funding acquisition, Data curation. **Zidong Zhu:** Writing – original draft, Visualization, Validation. **Yong Fan:** Writing – original draft, Software, Formal analysis, Conceptualization. **Jinghong Hu:** Data curation. **Jiaqi Wu:** Methodology. **Ziying Mu:** Software. **Yang Li:** Writing – review & editing, Validation, Supervision. **Qin Wei:** Writing – review & editing, Supervision, Funding acquisition, Conceptualization. **Chunmei Yang:** Project administration, Writing – review & editing. **Jing Tian:** Supervision, Investigation, Formal analysis, Data curation. **Shouqian Li:** Resources.

## **Declaration of competing interest**

The authors declare that they have no known competing financial interests or personal relationships that could have appeared to influence the work.

## Data Availability

Data will be made available on request.

## Appendix A Supplementary data

Supplementary material Image 1
