# Supplementary material for: Integrated microbiomic and metabolomic dynamics of Yi traditional fermented liquor
Source: Food Chem X. 2024 Nov 16;24:102016. doi: 10.1016/j.fochx.2024.102016 (PMC11629247; doi:10.1016/j.fochx.2024.102016)
Supplement: Supplementary file 1 — Supplementary material [file mmc1.docx]

Table S1. Parameters for GC-TOF-MS

| **Parameters** | **Value** |
| --- | --- |
| Sample Volume | 1 μL |
| Front Inlet Mode | Splitless Mode |
| Front Inlet Septum Purge Flow | 3 mL min^−1^ |
| Carrier Gas | Helium |
| Column | DB-5MS（30 m×250 μm×0.25 μm） |
| Column Flow | 1 mL min^−1^ |
| Oven Temperature Ramp | 50 °C hold on 1 min, raised to 310 °C at a rate of 10 °C min^−1^, hold on 8 min |
| Front Injection Temperature | 280 °C |
| Transfer Line Temperature | 280 °C |
| Ion Source Temperature | 250 °C |
| Electron Energy | -70 eV |
| Mass Range | m/z:50-500 |
| Acquisition Rate | 12.5 spectra per second |
| Solvent Delay | 6.25 min |

Table S2. Mobile Phase Gradient Table

| **Time flow rate (minutes)** | **A% H_2_O** | **B% Acetonitrile** |
| --- | --- | --- |
| 0 | 98 | 2 |
| 0.5 | 98 | 2 |
| 10 | 50 | 50 |
| 11 | 5 | 95 |
| 13 | 5 | 95 |
| 13.1 | 98 | 2 |
| 15 | 98 | 2 |

**Table S3. Results of Active Acidity Measurement**

| **Fermentation Time (Months)** | **pH Value** | **Titratable Acid (g/100mL)** | **Soluble Solids Content (%)** | **DPPH Radical**  **Scavenge Rate (%)** |
| --- | --- | --- | --- | --- |
| 1 | 3.69±0.006^b^ | 1.1122±0.0724^b^ | 25.23±0.15^abc^ | 45.86±3.08^b^ |
| 2 | 3.65±0.010^c^ | 1.3062±0.0126^a^ | 23.10±0.00^c^ | 39.44±2.33^bc^ |
| 3 | 3.66±0.029^c^ | 1.2969±0.0089^a^ | 24.47±0.75^bc^ | 43.00±0.73^b^ |
| 4 | 3.71±0.000^ab^ | 1.3115±0.0397^a^ | 26.07±1.10^ab^ | 34.62±1.85^e^ |
| 6 | 3.71±0.006^ab^ | 1.3028±0.0234^a^ | 23.07±0.23^c^ | 51.20±0.89^a^ |
| 8 | 3.74±0.006^a^ | 1.2950±0.0270^a^ | 26.13±0.61^a^ | 34.63±3.62^e^ |
| 10 | 3.76±0.000^a^ | 1.2163±0.0458^ab^ | 21.07±1.01^d^ | 37.60±1.48^d^ |

Table S4 Results of bacterial diversity analysis in the fermentation process of medicinal wine

| Month | Sample | Diversity index | | | Coverage% |
| --- | --- | --- | --- | --- | --- |
|  |  | Chao1 | Shannon | Simpson |  |
| 1st | A | 790.442±98.988ab | 2.858±0.847cd | 0.558±0.1cd | 99 |
| 2nd | B | 885.738±192.976a | 3.368±0.536bc | 0.65±0.098bc | 99 |
| 3th | C | 556.718±268.163c | 4.982±0.318a | 0.906±0.037a | 99 |
| 4th | D | 599.616±89.592bc | 1.774±0.632e | 0.41±0.132e | 99 |
| 6th | E | 817.402±158.395ab | 3.904±0.726b | 0.736±0.105b | 99 |
| 8th | F | 798.558±142.12ab | 3.55±0.368bc | 0.718±0.05b | 99 |
| 10th | G | 273.526±59.736d | 2.164±0.745de | 0.49±0.135de | 100 |

Table S5 Results of fungal diversity analysis in the process of fermentation

| Month | Sample | Diversity index | | | Coverage% |
| --- | --- | --- | --- | --- | --- |
|  |  | Chao1 | Shannon | Simpson |  |
| 1st | A | 940.65±216.36^b^ | 2.59±1.92^bc^ | 0.5±0.26^b^ | 99 |
| 2nd | B | 1302.29±234.79^a^ | 4.65±0.73^a^ | 0.86±0.06^a^ | 99 |
| 3th | C | 489.60±111.31^c^ | 1.83±0.49^cd^ | 0.56±0.05^b^ | 99.8 |
| 4th | D | 363.38±61.21^c^ | 1.5±0.46^cd^ | 0.54±0.05^b^ | 100 |
| 6th | E | 135.87±59.85^d^ | 1.12±0.08^d^ | 0.5±0.01^b^ | 100 |
| 8th | F | 433.62±146.65^c^ | 1.59±0.18^cd^ | 0.55±0.02^b^ | 99.8 |
| 10th | G | 448.37±79.85^c^ | 3.52±1.13^ab^ | 0.76±0.1^a^ | 100 |

**Table S6 Results of differential metabolite**

| Name | VIP | p | FC | CLASS | category | Which-Max | Name | VIP | p | FC | CLASS | category | Which-Max |
| --- | --- | --- | --- | --- | --- | --- | --- | --- | --- | --- | --- | --- | --- |
| Deethylatrazine | 1.255 | 0.105 | 16.278 | Alkaloids | AB | B | 16(R)-HETE | 1.425 | 0.039 | 5.135 | Lipids | AB | B |
| Ethyl 3,4,5-trimethoxybenzoate | 1.638 | 0.019 | 14.377 | Phenols | AB | B | Tropic acid | 0.838 | 0.267 | 4.969 | Phenols | AB | B |
| 5'-S-Methyl-5'-thioadenosine | 2.127 | 0.000 | 10.180 | Nucleotide And Its Derivates | AB | B | Ferulic acid | 1.973 | 0.000 | 4.398 | Phenylpropanoids | AB | B |
| N6-isopentenyladenosine | 1.978 | 0.000 | 9.709 | Nucleotide And Its Derivates | AB | B | Demethoxycurcumin | 2.011 | 0.000 | 4.278 | Phenols | AB | B |
| Diethylthiophosphate | 1.512 | 0.046 | 9.588 | Others | AB | B | 3-Methylindole | 1.052 | 0.165 | 4.063 | Alkaloids | AB | B |
| Phloretic acid | 0.930 | 0.233 | 9.544 | Phenols | AB | B | CYS-GLY | 1.725 | 0.005 | 3.939 | Amino Acid And Derivatives | AB | B |
| Docosahexaenoic acid | 1.577 | 0.021 | 9.361 | Lipids | AB | B | Isobergapten | 1.179 | 0.138 | 3.792 | Coumarins | AB | B |
| Homoorientin | 1.348 | 0.066 | 8.739 | Flavonoids | AB | B | Psychosine | 1.443 | 0.033 | 3.604 | Sugars | AB | B |
| Crotonoside | 0.941 | 0.229 | 8.667 | Alkaloids | AB | B | Furanodiene | 2.040 | 0.000 | 3.591 | Sesquiterpenoids | AB | B |
| Vitexin 2''-glucoside | 0.953 | 0.209 | 6.686 | Flavonoids | AB | B | Cyanidin-3-O-rhamnoside chloride | 1.763 | 0.006 | 3.567 | Flavonoids | AB | B |
| Indoleacetaldehyde | 1.864 | 0.002 | 6.669 | Alkaloids | AB | B | Bovinic acid | 1.755 | 0.011 | 3.554 | Lipids | AB | B |
| Ganoderic acid Jb | 1.388 | 0.067 | 6.469 | Terpenes | AB | B | Limonin | 1.032 | 0.160 | 3.536 | Terpenes | AB | B |
| Diosmin | 1.857 | 0.007 | 6.286 | Flavonoids | AB | B | Osajin | 1.515 | 0.023 | 3.364 | Flavonoids | AB | B |
| Saponarin | 1.859 | 0.007 | 6.233 | Flavonoids | AB | B | Cinnamic acid | 1.281 | 0.083 | 3.363 | Organic Acids And Derivatives | AB | B |
| Adenosine diphosphate ribose | 1.857 | 0.003 | 6.027 | Nucleotide And Its Derivates | AB | B | Cynarin | 0.661 | 0.380 | 3.162 | Organic Acids And Derivatives | AB | B |
| Narcissoside | 1.074 | 0.145 | 5.271 | Flavonoids | AB | B | Phloretin | 1.578 | 0.030 | 3.148 | Flavonoids | AB | B |
| Podophyllotoxinone | 1.500 | 0.046 | 5.210 | Lignans | AB | B | 3-Aminoisobutanoic acid | 0.541 | 0.484 | 3.130 | Amino Acid And Derivatives | AB | B |
| Scopolamine | 0.938 | 0.193 | 3.046 | Alkaloids | AB | B | L-Methionine | 1.397 | 0.074 | 2.589 | Amino Acid And Derivatives | AB | B |
| Guanosine | 1.990 | 0.000 | 3.035 | Nucleotide And Its Derivates | AB | B | Dihydrosanguinarine | 1.707 | 0.014 | 2.583 | Alkaloids | AB | B |
| Neohesperidin | 1.182 | 0.131 | 3.034 | Flavonoids | AB | B | Phytic acid | 1.294 | 0.066 | 2.575 | Organic Acids And Derivatives | AB | B |
| 4-Pyridoxolactone | 1.616 | 0.015 | 3.029 | Alkaloids | AB | B | Salidroside | 0.918 | 0.213 | 2.549 | Phenols | AB | B |
| 4-Methylcatechol | 1.114 | 0.153 | 2.947 | Phenols | AB | B | Alliin | 2.016 | 0.000 | 2.538 | Others | AB | B |
| Sedoheptulose | 1.763 | 0.009 | 2.888 | Sugars | AB | B | Nordihydroguaiaretic acid | 1.975 | 0.001 | 2.536 | Lignans | AB | B |
| Homoeriodictyol | 1.939 | 0.002 | 2.869 | Flavonoids | AB | B | Phyllalbine | 1.750 | 0.006 | 2.535 | Alkaloids | AB | B |
| Peonidin-3-glucoside | 1.939 | 0.001 | 2.860 | Flavonoids | AB | B | Hesperidin | 1.797 | 0.005 | 2.524 | Flavonoids | AB | B |
| Guanine | 2.048 | 0.000 | 2.843 | Nucleotide And Its Derivates | AB | B | Estriol | 1.266 | 0.084 | 2.499 | Steroids | AB | B |
| Leucodin | 1.928 | 0.002 | 2.823 | Sesquiterpenoids | AB | B | Sipeimine | 1.196 | 0.095 | 2.464 | Alkaloids | AB | B |
| Cyanidin 3-rutinoside | 2.055 | 0.000 | 2.811 | Flavonoids | AB | B | Norlichexanthone | 1.878 | 0.004 | 2.351 | Quinones | AB | B |
| Arachidonic acid | 1.380 | 0.074 | 2.784 | Lipids | AB | B | Delphinidin-3-O-glucoside | 1.282 | 0.105 | 2.315 | Flavonoids | AB | B |
| o-Cresol | 1.267 | 0.074 | 2.784 | Phenols | AB | B | Isoquercitrin | 1.282 | 0.105 | 2.315 | Flavonoids | AB | B |
| N1-Methyl-4-pyridone-3-carboxamide | 1.921 | 0.003 | 2.769 | Alkaloids | AB | B | Buddlenoid A | 1.013 | 0.172 | 2.314 | Flavonoids | AB | B |
| Troxeruti | 1.389 | 0.050 | 2.767 | Flavonoids | AB | B | Protocatechualdehyde | 1.823 | 0.003 | 2.299 | Flavonoids | AB | B |
| Cymarin | 1.082 | 0.136 | 2.749 | Steroids | AB | B | Rutin | 2.066 | 0.000 | 2.272 | Flavonoids | AB | B |
| Robinetin | 1.909 | 0.001 | 2.735 | Flavonoids | AB | B | 5-Hydroxymethyluracil | 1.670 | 0.007 | 2.270 | Nucleotide And Its Derivates | AB | B |
| Cleomiscosin A | 1.966 | 0.000 | 2.664 | Coumarins | AB | B | Oleocanthal | 1.380 | 0.058 | 2.248 | Phenols | AB | B |
| Pachymic acid | 1.427 | 0.035 | 2.659 | Terpenes | AB | B | Liquiritin | 1.404 | 0.043 | 2.241 | Flavonoids | AB | B |
| Raffinose | 0.760 | 0.318 | 2.627 | Sugars | AB | B | Pristimerin | 0.944 | 0.194 | 2.196 | Terpenes | AB | B |
| Angelicin | 2.090 | 0.000 | 2.622 | Phenylpropanoids | AB | B | 4-Sulfobenzoate | 1.556 | 0.019 | 2.188 | Organic Acids And Derivatives | AB | B |
| Lucidumol A | 1.494 | 0.048 | 2.616 | Terpenes | AB | B | Kaempferol 3-O-beta-sophoroside | 1.493 | 0.025 | 2.175 | Flavonoids | AB | B |
| Rhoifolin | 2.042 | 0.000 | 2.609 | Flavonoids | AB | B | 9-Methoxy-alpha-lapachone | 1.603 | 0.013 | 2.172 | Quinones | AB | B |
| Neoeriocitrin | 1.540 | 0.020 | 2.162 | Flavonoids | AB | B | Curcolone | 1.158 | 0.107 | 2.038 | Sesquiterpenoids | AB | B |
| Linarin | 0.120 | 0.920 | 2.148 | Flavonoids | AB | B | Xanthatin | 1.158 | 0.107 | 2.038 | Sesquiterpenoids | AB | B |
| Beta-Sitosterol | 1.314 | 0.076 | 2.116 | Steroids | AB | B | Retinoic acid | 1.477 | 0.041 | 2.029 | Terpenes | AB | B |
| Gallic acid | 0.812 | 0.272 | 2.093 | Phenols | AB | B | Cryptomeridiol 11-rhamnoside | 1.092 | 0.159 | 0.181 | Sesquiterpenoids | AB | A |
| 8-Geranyloxypsoralen | 1.265 | 0.079 | 2.087 | Coumarins | AB | B | Alexine | 1.058 | 0.167 | 0.167 | Alkaloids | AB | A |
| Dihydrorobinetin | 1.242 | 0.110 | 2.081 | Flavonoids | AB | B | Phenethylamine | 1.054 | 0.170 | 0.093 | Alkaloids | AB | A |
| Arctiin | 1.390 | 0.068 | 2.072 | Lignans | AB | B | Tacrolimus | 0.975 | 0.219 | 0.079 | Alkaloids | AB | A |
| Adenosine | 0.653 | 0.393 | 2.055 | Nucleotide And Its Derivates | AB | B | Porson | 1.572 | 0.029 | 0.065 | Phenols | AB | A |
| Carbendazim | 1.432 | 0.038 | 2.053 | Alkaloids | AB | B | Indole | 0.618 | 0.445 | 0.062 | Alkaloids | AB | A |
| Micromelin | 1.641 | 0.010 | 2.042 | Coumarins | AB | B | p-Octopamine | 1.332 | 0.088 | 0.001 | Phenols | AB | A |
| p-Octopamine | 1.210 | 0.141 | 585.130 | Phenols | BC | C | Colchicine | 1.957 | 0.005 | 4.588 | Alkaloids | BC | C |
| Indole | 1.150 | 0.171 | 24.833 | Alkaloids | BC | C | 11(R)-HETE | 1.491 | 0.089 | 4.285 | Lipids | BC | C |
| Tacrolimus | 1.111 | 0.181 | 20.586 | Alkaloids | BC | C | Malvidin 3-O-glucoside (Oenin) | 1.470 | 0.073 | 4.141 | Flavonoids | BC | C |
| Soyacerebroside I | 2.310 | 0.000 | 13.827 | Sugars | BC | C | Saccharate | 1.394 | 0.086 | 3.979 | Sugars | BC | C |
| Xanthotoxin | 1.590 | 0.062 | 13.812 | Phenylpropanoids | BC | C | Adenine | 1.757 | 0.017 | 3.718 | Nucleotide And Its Derivates | BC | C |
| Phenethylamine | 0.999 | 0.271 | 11.793 | Alkaloids | BC | C | Ferulic acid | 1.489 | 0.073 | 3.639 | Phenylpropanoids | BC | C |
| Procyanidin B2 | 1.340 | 0.125 | 8.938 | Flavonoids | BC | C | Inositol | 1.625 | 0.035 | 3.432 | Alcohols And Polyols | BC | C |
| Procyanidin B1 | 0.794 | 0.334 | 7.258 | Flavonoids | BC | C | N6-isopentenyladenosine | 0.860 | 0.316 | 3.376 | Nucleotide And Its Derivates | BC | C |
| CYS-GLY | 1.517 | 0.060 | 6.620 | Amino Acid And Derivatives | BC | C | Alexine | 1.664 | 0.027 | 3.259 | Alkaloids | BC | C |
| Argininosuccinate | 1.269 | 0.128 | 2.877 | Organic Acids And Derivatives | BC | C | Galantamine | 1.388 | 0.088 | 2.314 | Alkaloids | BC | C |
| Daidzin | 1.586 | 0.032 | 2.855 | Flavonoids | BC | C | 3-Galloylquinic acid | 1.688 | 0.026 | 2.311 | Phenols | BC | C |
| Adenosine diphosphate ribose | 0.650 | 0.458 | 2.821 | Nucleotide And Its Derivates | BC | C | Tropic acid | 0.786 | 0.330 | 2.306 | Phenols | BC | C |
| Adenosine 3'-monophosphate | 1.677 | 0.032 | 2.665 | Nucleotide And Its Derivates | BC | C | Adenosine 5'-monophosphate | 1.524 | 0.060 | 2.304 | Nucleotide And Its Derivates | BC | C |
| Cauloside A | 1.241 | 0.124 | 2.613 | Terpenes | BC | C | D-Xylulose | 0.925 | 0.284 | 2.303 | Sugars | BC | C |
| Narcissoside | 1.207 | 0.152 | 2.606 | Flavonoids | BC | C | Swertiajaponin | 1.907 | 0.022 | 2.292 | Flavonoids | BC | C |
| Phenol | 0.314 | 0.628 | 2.604 | Phenols | BC | C | Abietic acid | 0.872 | 0.293 | 2.281 | Terpenes | BC | C |
| Pyrogallol | 1.779 | 0.036 | 2.590 | Phenols | BC | C | 7-Ethoxycoumarin | 1.908 | 0.014 | 2.264 | Coumarins | BC | C |
| N-((-)-jasmonoyl)-S-isoleucine | 1.588 | 0.036 | 2.590 | Amino Acid And Derivatives | BC | C | Sophoricoside | 0.349 | 0.749 | 2.206 | Flavonoids | BC | C |
| Ginsenoside Rf | 1.146 | 0.188 | 2.554 | Terpenes | BC | C | Officinalisinin I | 1.656 | 0.039 | 2.156 | Steroids | BC | C |
| Sesamolin | 2.048 | 0.004 | 2.503 | Lignans | BC | C | Hydroxysafflor yellow A | 0.851 | 0.324 | 2.156 | Flavonoids | BC | C |
| 1-Caffeoylquinic acid | 1.336 | 0.104 | 2.501 | Phenylpropanoids | BC | C | Rubusoside | 0.867 | 0.297 | 2.149 | Terpenes | BC | C |
| 2-Methylbenzaldehyde | 2.070 | 0.003 | 2.487 | Benzene And Substituted Derivatives | BC | C | Acetovanillone | 2.172 | 0.002 | 2.147 | Phenols | BC | C |
| L-2-Hydroxyglutaric acid | 0.810 | 0.327 | 2.455 | Lipids | BC | C | Quercetin 3-O-neohesperidoside | 1.440 | 0.068 | 2.135 | Flavonoids | BC | C |
| Chalconaringenin | 0.680 | 0.387 | 2.453 | Flavonoids | BC | C | Apiin | 1.474 | 0.077 | 2.134 | Flavonoids | BC | C |
| 4-Methylumbelliferone | 1.805 | 0.016 | 2.416 | Coumarins | BC | C | Isobergapten | 1.449 | 0.084 | 2.130 | Coumarins | BC | C |
| Malvidin-3-O-galactoside | 1.427 | 0.072 | 2.399 | Flavonoids | BC | C | Capsanthin | 1.744 | 0.025 | 2.114 | Terpenes | BC | C |
| 4-Methoxyphenyl beta-D-glucopyranoside | 1.894 | 0.007 | 2.347 | Phenols | BC | C | Mangiferin | 1.712 | 0.025 | 2.114 | Quinones | BC | C |
| Homoferreirin | 1.760 | 0.016 | 2.341 | Flavonoids | BC | C | Adenosine | 0.169 | 0.915 | 2.099 | Nucleotide And Its Derivates | BC | C |
| Demethoxycurcumin | 1.090 | 0.212 | 2.098 | Phenols | BC | C | N-Methyltyramine | 1.638 | 0.034 | 2.046 | Alkaloids | BC | C |
| Knightinol | 1.536 | 0.052 | 2.095 | Alkaloids | BC | C | Buddlenoid A | 1.065 | 0.211 | 2.042 | Flavonoids | BC | C |
| 3-Hydroxy-4-methoxycinnamic acid | 1.097 | 0.219 | 2.071 | Phenylpropanoids | BC | C | Homoeriodictyol | 1.994 | 0.021 | 0.167 | Flavonoids | BC | B |
| 1-Isomangostin | 1.984 | 0.005 | 2.041 | Quinones | BC | C | Bovinic acid | 2.352 | 0.000 | 0.146 | Lipids | BC | B |
| Okanin | 0.999 | 0.232 | 2.009 | Flavonoids | BC | C | 16(R)-HETE | 1.870 | 0.016 | 0.117 | Lipids | BC | B |
| (-)-Epigallocatechin | 2.078 | 0.002 | 2.008 | Flavonoids | BC | C | Phloretic acid | 1.077 | 0.231 | 0.108 | Phenols | BC | B |
| Cyclocurcumin | 0.387 | 0.633 | 2.002 | Phenols | BC | C | Gallic acid | 1.321 | 0.137 | 0.094 | Phenols | BC | B |
| 3-Aminoisobutanoic acid | 0.907 | 0.268 | 2.001 | Amino Acid And Derivatives | BC | C |  |  |  |  |  |  |  |
| Adenosine 5'-monophosphate | 0.809 | 0.342 | 6.166 | Nucleotide And Its Derivates | CD | D | Cimifugin | 1.984 | 0.004 | 2.826 | Flavonoids | CD | D |
| Amentoflavone | 1.973 | 0.002 | 5.432 | Flavonoids | CD | D | Pomiferin | 0.954 | 0.241 | 2.817 | Flavonoids | CD | D |
| C-Veratroylglycol | 1.558 | 0.035 | 5.405 | Phenylpropanoids | CD | D | 1-(4-Hydroxyphenyl)propan-1-one | 1.079 | 0.173 | 2.708 | Phenylpropanoids | CD | D |
| Alpha-Terpineol | 1.962 | 0.004 | 4.012 | Terpenes | CD | D | Traumatic acid | 0.220 | 0.792 | 2.695 | Lipids | CD | D |
| Karacoline | 1.734 | 0.022 | 3.952 | Alkaloids | CD | D | Ethyl 3,4,5-trimethoxybenzoate | 0.129 | 0.826 | 2.684 | Phenols | CD | D |
| Cinnamic acid | 1.074 | 0.193 | 3.837 | Organic Acids And Derivatives | CD | D | Lipiferolide | 1.513 | 0.047 | 2.610 | Sesquiterpenoids | CD | D |
| 1-Naphthol | 1.558 | 0.047 | 3.755 | Alcohols And Polyols | CD | D | 4-Methylcatechol | 0.794 | 0.345 | 2.538 | Phenols | CD | D |
| Nicotinurate | 0.697 | 0.386 | 3.753 | Alkaloids | CD | D | S-Lactoylglutathione | 0.934 | 0.233 | 2.484 | Amino Acid And Derivatives | CD | D |
| Anabasine | 1.437 | 0.099 | 3.406 | Alkaloids | CD | D | Eriodictyol | 1.296 | 0.125 | 2.462 | Flavonoids | CD | D |
| Ganoderic acid Jb | 1.121 | 0.148 | 3.296 | Terpenes | CD | D | vitamin K2 | 1.886 | 0.012 | 2.434 | Vitamins | CD | D |
| Cedrelone | 1.055 | 0.201 | 2.939 | Terpenes | CD | D | Guanine | 1.834 | 0.011 | 0.190 | Nucleotide And Its Derivates | CD | C |
| Swertiamarin | 2.030 | 0.001 | 2.878 | Terpenes | CD | D | N1-Methyl-4-pyridone-3-carboxamide | 1.984 | 0.004 | 0.188 | Alkaloids | CD | C |
| 2-Hydroxy-3-carboxybenzalpyruvate | 1.133 | 0.151 | 2.839 | Others | CD | D | Isobergapten | 1.420 | 0.100 | 0.187 | Coumarins | CD | C |
| Bovinic acid | 1.289 | 0.113 | 2.198 | Lipids | CD | D | Saccharate | 1.526 | 0.035 | 0.184 | Sugars | CD | C |
| Hydroquinone | 1.842 | 0.015 | 2.191 | Phenols | CD | D | Buddlenoid A | 1.910 | 0.009 | 0.179 | Flavonoids | CD | C |
| Ephedrine | 1.564 | 0.036 | 2.178 | Benzene And Substituted Derivatives | CD | D | Tacrolimus | 0.751 | 0.375 | 0.176 | Alkaloids | CD | C |
| Practolol | 0.815 | 0.413 | 2.139 | Benzene And Substituted Derivatives | CD | D | Homoorientin | 1.134 | 0.179 | 0.173 | Flavonoids | CD | C |
| Lactaroviolin | 0.972 | 0.219 | 2.072 | Lipids | CD | D | Guanosine | 1.805 | 0.020 | 0.156 | Nucleotide And Its Derivates | CD | C |
| Tenulin | 1.881 | 0.006 | 2.070 | Sesquiterpenoids | CD | D | Ferulic acid | 1.816 | 0.011 | 0.139 | Phenylpropanoids | CD | C |
| Pinocembrin | 1.044 | 0.214 | 2.069 | Flavonoids | CD | D | Narcissoside | 1.782 | 0.012 | 0.121 | Flavonoids | CD | C |
| Evoxine | 0.547 | 0.483 | 2.059 | Alkaloids | CD | D | Procyanidin B2 | 1.263 | 0.116 | 0.112 | Flavonoids | CD | C |
| Curryangine | 1.597 | 0.054 | 2.046 | Alkaloids | CD | D | CYS-GLY | 1.649 | 0.033 | 0.105 | Amino Acid And Derivatives | CD | C |
| 3-Hydroxy-2-methylpyridine | 1.715 | 0.033 | 2.016 | Alkaloids | CD | D | Xanthotoxin | 1.438 | 0.065 | 0.085 | Phenylpropanoids | CD | C |
| Homoeriodictyol | 1.563 | 0.074 | 2.369 | Flavonoids | CD | D | Adenosine diphosphate ribose | 1.631 | 0.026 | 0.072 | Nucleotide And Its Derivates | CD | C |
| Phytanic acid | 1.266 | 0.128 | 2.320 | Lipids | CD | D | N6-isopentenyladenosine | 1.808 | 0.016 | 0.049 | Nucleotide And Its Derivates | CD | C |
| Gamabufotalin | 1.799 | 0.013 | 2.247 | Steroids | CD | D | p-Octopamine | 1.207 | 0.139 | 0.002 | Phenols | CD | C |
| p-Octopamine | 0.913 | 0.366 | 464.300 | Phenols | DG | G | Phloretic acid | 0.864 | 0.384 | 7.675 | Phenols | DG | G |
| Gallic acid | 1.028 | 0.312 | 37.746 | Phenols | DG | G | Sciadopitysin | 1.716 | 0.061 | 2.315 | Flavonoids | DG | G |
| Guanosine | 2.679 | 0.000 | 2.490 | Nucleotide And Its Derivates | DG | G | Poncirin | 2.000 | 0.026 | 2.305 | Flavonoids | DG | G |
| N6-isopentenyladenosine | 2.660 | 0.000 | 6.858 | Nucleotide And Its Derivates | DG | G | Linarin | 1.923 | 0.056 | 2.293 | Flavonoids | DG | G |
| Cryptomeridiol 11-rhamnoside | 1.688 | 0.074 | 4.646 | Sesquiterpenoids | DG | G | Lumichrome | 0.205 | 0.858 | 2.292 | Alkaloids | DG | G |
| Crotonoside | 1.086 | 0.273 | 4.587 | Alkaloids | DG | G | Folic acid | 1.767 | 0.056 | 2.288 | Alkaloids | DG | G |
| pyrocatechol | 1.389 | 0.168 | 4.454 | Flavonoids | DG | G | Guanine | 2.468 | 0.001 | 2.262 | Nucleotide And Its Derivates | DG | G |
| Sophoricoside | 1.064 | 0.319 | 4.364 | Flavonoids | DG | G | Flavin adenine dinucleotide (FAD) | 1.305 | 0.175 | 2.253 | Nucleotide And Its Derivates | DG | G |
| Procyanidin B2 | 1.377 | 0.162 | 4.266 | Flavonoids | DG | G | Diacetoxy-6-gingerdiol | 0.689 | 0.509 | 2.243 | Phenols | DG | G |
| Adenosine diphosphate ribose | 2.287 | 0.011 | 4.174 | Nucleotide And Its Derivates | DG | G | NADP | 1.277 | 0.202 | 2.232 | Nucleotide And Its Derivates | DG | G |
| Tectorigenin | 0.204 | 0.902 | 4.045 | Flavonoids | DG | G | Uncinatone | 0.381 | 0.713 | 2.202 | Terpenes | DG | G |
| Salidroside | 1.652 | 0.076 | 3.680 | Phenols | DG | G | k-Strophanthoside | 1.390 | 0.162 | 2.187 | Steroids | DG | G |
| Narcissoside | 1.933 | 0.047 | 3.408 | Flavonoids | DG | G | 3,4,5-Trimethoxycinnamyl alcohol | 1.547 | 0.102 | 2.168 | Phenylpropanoids | DG | G |
| Crocin | 2.043 | 0.033 | 3.191 | Terpenes | DG | G | Isobergapten | 1.315 | 0.212 | 2.146 | Coumarins | DG | G |
| Isorhamnetin | 1.206 | 0.208 | 2.761 | Flavonoids | DG | G | Ferulic acid | 2.225 | 0.012 | 2.138 | Phenylpropanoids | DG | G |
| 8-Gingerol | 1.234 | 0.232 | 2.748 | Phenols | DG | G | Auriculine | 0.419 | 0.677 | 2.109 | Alkaloids | DG | G |
| Sulfuretin | 1.807 | 0.058 | 2.734 | Flavonoids | DG | G | CYS-GLY | 2.206 | 0.010 | 2.093 | Amino Acid And Derivatives | DG | G |
| Diosbulbin C | 1.058 | 0.289 | 2.632 | Terpenes | DG | G | Quercitrin | 1.574 | 0.122 | 2.083 | Flavonoids | DG | G |
| Ginsenoside Rf | 1.391 | 0.148 | 2.552 | Terpenes | DG | G | Buddlenoid A | 1.764 | 0.060 | 2.022 | Flavonoids | DG | G |
| N-Methylcytisine | 1.660 | 0.075 | 2.548 | Alkaloids | DG | G | Vanillic acid | 1.462 | 0.146 | 2.008 | Phenols | DG | G |
| Tacrolimus | 0.065 | 0.988 | 2.528 | Alkaloids | DG | G | Glycitin | 2.076 | 0.016 | 0.192 | Flavonoids | DG | D |
| indolin-2-one | 0.982 | 0.344 | 2.430 | Alkaloids | DG | G | 5-Methyldeoxycytidine | 1.665 | 0.063 | 0.141 | Nucleotide And Its Derivates | DG | D |
| Carbendazim | 0.563 | 0.584 | 2.417 | Alkaloids | DG | G |  |  |  |  |  |  |  |
